# From Tradition to Innovation: Diverse Molecular Techniques in the Fight Against Infectious Diseases

**DOI:** 10.3390/diagnostics14242876

**Published:** 2024-12-21

**Authors:** Ahmed Nouri Alsharksi, Serhat Sirekbasan, Tuğba Gürkök-Tan, Adam Mustapha

**Affiliations:** 1 Department of Microbiology, Faculty of Medicine, Misurata University, Misrata 93FH+66F, Libya; ahmedalsherkas87@gmail.com; 2 Department of Medical Laboratory Techniques, Şabanözü Vocational School, Çankırı Karatekin University, Çankırı 18650, Turkey; 3 Department of Field Crops, Food and Agriculture Vocational School, Çankırı Karatekin University, Çankırı 18100, Turkey; t.gurkok@karatekin.edu.tr; 4 Department of Microbiology, Faculty of Life Sciences, University of Maiduguri, Maiduguri 600104, Nigeria; adadmustapha@unimaid.edu.ng

**Keywords:** infectious diseases, molecular diagnostics, next-generation sequencing (NGS), polymerase chain reaction (PCR)

## Abstract

Infectious diseases impose a significant burden on global health systems due to high morbidity and mortality rates. According to the World Health Organization, millions die from infectious diseases annually, often due to delays in accurate diagnosis. Traditional diagnostic methods in clinical microbiology, primarily culture-based techniques, are time-consuming and may fail with hard-to-culture pathogens. Molecular biology advancements, notably the polymerase chain reaction (PCR), have revolutionized infectious disease diagnostics by allowing rapid and sensitive detection of pathogens’ genetic material. PCR has become the gold standard for many infections, particularly highlighted during the COVID-19 pandemic. Following PCR, next-generation sequencing (NGS) has emerged, enabling comprehensive genomic analysis of pathogens, thus facilitating the detection of new strains and antibiotic resistance tracking. Innovative approaches like CRISPR technology are also enhancing diagnostic precision by identifying specific DNA/RNA sequences. However, the implementation of these methods faces challenges, particularly in low- and middle-income countries due to infrastructural and financial constraints. This review will explore the role of molecular diagnostic methods in infectious disease diagnosis, comparing their advantages and limitations, with a focus on PCR and NGS technologies and their future potential.

## 1. Introduction

Infectious diseases pose a significant burden on health systems worldwide due to high morbidity and mortality rates. According to the World Health Organization (WHO), millions of people die from infectious diseases each year, often because early and accurate diagnosis is not achieved [1]. This reality highlights the critical importance of rapid and accurate diagnosis of infectious diseases.

For many years, traditional diagnostic methods used in clinical microbiology laboratories were generally based on culture-based techniques. However, these techniques are not only time-consuming but also inadequate for pathogens that are difficult or impossible to cultivate under laboratory conditions [2]. This limitation is particularly evident in the case of slow-growing microorganisms or pathogens requiring highly specific culture conditions. Additionally, the sensitivity of indirect immunological tests (serology) may significantly decrease during the early stages of infection, when specific antibody formation has not yet occurred [3].

In recent years, advancements in molecular biology have led to significant transformations in the diagnosis of infectious diseases, ushering in a new era in diagnostic processes. Among these advancements, the polymerase chain reaction (PCR), developed by Kary Mullis in the late 1980s, stands out [4]. The primary advantage of PCR is its ability to rapidly and sensitively amplify the genetic material of the infectious agent, enabling specific detection. This capability has quickly made PCR an indispensable tool in clinical microbiology laboratories and established it as the gold standard for diagnosing many infectious diseases. During the COVID-19 pandemic, PCR technology became the most widely used diagnostic method worldwide [5,6]. The rapid and reliable results provided by PCR-based tests during the pandemic have once again highlighted the critical importance of molecular diagnostic methods for public health and clinical practice.

One of the most remarkable innovations following PCR is next-generation sequencing (NGS) technology. NGS allows for the sequencing of entire genomes of pathogens in a single run, enabling the detection of new strains and genetic variations [7]. This technology offers significant advantages, particularly in tracking antibiotic-resistant strains, identifying virulence factors, and understanding the molecular epidemiology of outbreaks. With the growing availability of bioinformatics tools and data analysis capabilities, NGS is increasingly being used in clinical diagnostics. The rapid identification of resistance genes and virulence factors has greatly accelerated the management of infections [8].

Molecular diagnostic methods have expanded beyond PCR and NGS to include innovative approaches such as CRISPR-based diagnostic systems. In addition to its gene-editing potential, CRISPR (short for “clustered regularly interspaced short palindromic repeats”) technology stands out as a precise and rapid method for detecting specific DNA or RNA sequences in the diagnosis of infectious diseases. This technology marks the beginning of a new era in the diagnosis of infectious diseases [9,10].

However, the implementation of molecular diagnostic methods in clinical practice presents certain challenges, especially in low- and middle-income countries. The lack of advanced infrastructure, trained personnel, and sufficient funding poses significant barriers to the widespread adoption of these technologies [11]. Nonetheless, ongoing efforts to develop portable and more affordable PCR and NGS systems may help overcome these challenges [12,13].

With the rapid advancement of molecular diagnostic methods, the advantages and limitations of each technology have become highly significant for clinical applications. Techniques such as PCR, NGS, loop-mediated isothermal amplification (LAMP), nucleic acid sequence-based amplification (NASBA), and CRISPR offer speed, sensitivity, and specificity in the diagnosis of infectious diseases, yet each has its own distinct strengths and weaknesses. Table 1 provides a comparative overview of the advantages, disadvantages, and applications of these technologies, offering guidance on which methods may be preferred under different conditions in molecular diagnostic processes.

This review will provide a detailed examination of the role of molecular diagnostic methods in the diagnosis of infectious diseases, how they are used in clinical applications, their advantages, and the challenges encountered. Particular emphasis will be placed on advanced diagnostic methods such as PCR and NGS, highlighting the advantages they offer over traditional methods and discussing potential future developments.

## 2. Molecular Diagnostic Methods

Molecular diagnostic methods are advanced biotechnological tools that enable the rapid, sensitive, and specific detection of infectious diseases. The slow nature and limited sensitivity of traditional diagnostic methods pose a significant disadvantage, especially for infections that require prompt intervention. In contrast, molecular methods target the genetic material (DNA and RNA) of pathogens, providing faster and more accurate results. The ability of these techniques to be used in the early stages of infection and to detect even low amounts of pathogenic material makes them indispensable in clinical applications [14,15].

Today, the most commonly used techniques in molecular diagnostics include PCR, Real-Time PCR (qPCR), Multiplex PCR, NGS, LAMP, and CRISPR-based diagnostic methods. These techniques not only accelerate diagnostic processes but also provide critical information about the genetic structure of infectious agents, contributing to treatment planning.

### 2.1. Polymerase Chain Reaction (PCR)

PCR is considered one of the cornerstones of molecular diagnostics and has been a revolutionary development in the diagnosis of infectious diseases. Developed by Kary Mullis in 1983, this technique allows for the rapid amplification of a specific segment of target DNA millions of times, enabling the detection of even extremely small amounts of genetic material [4]. This high sensitivity has made PCR an indispensable tool in the diagnosis of infectious diseases. Today, PCR is used to detect a wide range of microorganisms, including viruses, bacteria, fungi, and parasites, and is regarded as the “gold standard” in clinical microbiology laboratories [16].

**Figure 1:**
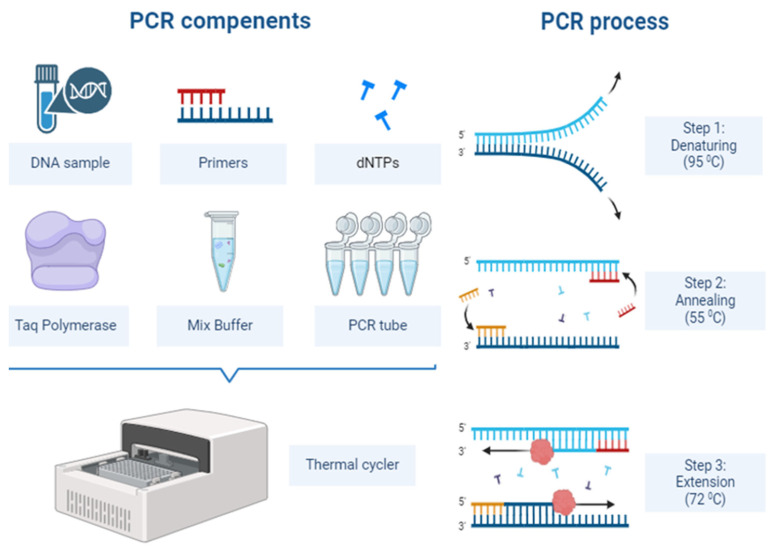
Working principle of polymerase chain reaction.

The principle of PCR involves targeting a specific region of DNA or RNA and amplifying this region using primers, allowing for the detection of the pathogen in a laboratory setting (Figure 1). One of the main reasons for the widespread use of this method in infectious diseases is its ability to detect even very small amounts of pathogenic DNA or RNA [17]. PCR provides highly sensitive and specific results, especially in the early stages of viral infections such as HIV, Hepatitis B (HBV), and Hepatitis C (HCV) [18,19]. Similarly, for bacterial infections like tuberculosis [20], *Legionella pneumophila* [21], and *Streptococcus pneumoniae*, PCR offers faster and more reliable results compared to traditional culture methods [22].

Over time, advanced variants of PCR have made significant progress in the diagnosis of infectious diseases. For example, Real-Time PCR (qPCR) performs DNA amplification while simultaneously measuring the amount of amplified DNA in real time. By using a fluorescent signal to monitor the amplification process, qPCR indicates how much target DNA is being replicated, allowing for both qualitative and quantitative analysis. This technology plays a crucial role in determining disease burden and monitoring response to treatment. qPCR offers high sensitivity and specificity, making it a powerful tool for pathogen detection [23]. During the COVID-19 pandemic, it was the most widely used method for detecting SARS-CoV-2 [24]. Additionally, because qPCR can detect multiple pathogens simultaneously, it plays an important role in identifying co-infections [25].

Multiplex PCR is a technique that enables the simultaneous amplification of multiple target DNA regions within a single reaction tube. This method is advantageous in detecting multiple pathogens or resistance genes from the same sample. Different technologies are used to distinguish amplification signals in multiplex PCR, such as molecular beacons and TaqMan probes, which provide specific fluorescent signals for each target sequence [26]. This approach is particularly advantageous in diagnosing respiratory infections, where multiple viruses or bacteria may coexist within the same patient sample [27]. For instance, the Unyvero HPN system (Curetis GmbH, Holzgerlingen, Germany) can detect 21 different bacteria and one parasite semi-quantitatively (from + to +++) and identify 15 resistance genes in approximately 5 h. This comprehensive detection capability provides clinicians with valuable information on co-infections and antimicrobial resistance patterns in a relatively short time [28]. Additionally, multiplex PCR can detect antimicrobial resistance genes at the same time, playing a crucial role in managing patient treatment [29].

Digital PCR (dPCR) is a newer and more advanced form of PCR, in which the sample is partitioned into thousands of micro-reactions to enhance quantitative accuracy. dPCR allows for the more sensitive and precise detection of nucleic acids present in low copy numbers. As a result, it is used for detecting rare mutations or low levels of pathogens [30].

Despite the widespread use of PCR in infectious diseases, it has some limitations. For instance, this method only indicates the presence of DNA or RNA, which does not always signify an active infection. Additionally, PCR testing is prone to technical errors that can result in false positive or negative results. Therefore, it is important to support PCR results with clinical findings.

#### 2.1.1. Applications of PCR in the Diagnosis of Infectious Diseases

PCR has become an indispensable tool in modern clinical microbiology laboratories due to its wide range of applications in diagnosing infectious diseases [31]. One of the major advantages of PCR is its ability to produce results quickly, with a typical PCR process completed within just a few hours, which is crucial for the rapid diagnosis of infections. Additionally, its high sensitivity allows for the detection of even very small amounts of pathogenic DNA, setting it apart from other diagnostic methods. It is widely used in the diagnosis of viral, bacterial, fungal, and parasitic infections, providing significant advantages in the clinical management of infections [17,32].

##### Detection of Viral Infections

PCR has been a groundbreaking method in the diagnosis of viral infections. Traditional serological methods can only yield positive results at specific stages of an infection, whereas PCR technology can detect the virus’s genetic material at much earlier stages, thereby speeding up the diagnostic process [33].

PCR technology is indispensable for the detection of HBV and HCV. The detection of HCV RNA using PCR can be performed in the early stages of infection, allowing for the rapid identification of these viruses, which have the potential to become chronic. Moreover, PCR enables the measurement of viral load, which is useful for monitoring the progression of the disease and the response to antiviral treatment [34,35].

PCR also plays a crucial role in the diagnosis of Human Immunodeficiency Virus (HIV) infection. It can detect the virus’s RNA even during the pre-seroconversion period, providing an opportunity to initiate antiretroviral therapy (ART) early on [36]. The quantitative applications of PCR are essential for monitoring viral load in HIV patients, allowing for rapid intervention in cases of treatment failure or disease progression [37].

During the COVID-19 pandemic, PCR became one of the most widely used methods for detecting SARS-CoV-2. The amplification of RNA from nasopharyngeal swab samples using reverse transcription PCR (RT-PCR) emerged as the most common approach for diagnosing the disease. The high sensitivity of RT-PCR allowed for the detection of not only symptomatic cases but also asymptomatic carriers, thus playing a significant role in controlling the spread of the pandemic [5,38,39].

##### Detection of Bacterial Infections

One of the main advantages of PCR in the diagnosis of bacterial infections is its ability to provide results much faster than traditional culture methods. The speed and accuracy offered by PCR are crucial for clinical outcomes, especially in serious bacterial infections that require rapid treatment [40].

Sepsis is one of the most serious examples of bacterial infections that require rapid intervention. PCR allows for the quick identification of pathogens causing sepsis, enabling the prompt initiation of appropriate antibiotic treatment. While diagnosis using traditional blood culture methods can take several days, PCR can complete the process within hours. This has a direct impact on the course of the disease and the patient’s chances of survival [41].

In the diagnosis of chronic bacterial infections such as tuberculosis (TB), the speed and sensitivity provided by PCR make a significant difference. While culturing *Mycobacterium tuberculosis*, the causative agent of tuberculosis, using traditional methods can take weeks, PCR can diagnose tuberculosis within a few hours [42,43]. Moreover, PCR has the ability to detect antibiotic resistance genes, making it an important guide in treatment planning. For instance, the rapid identification of strains resistant to drugs such as rifampicin and isoniazid allows for the swift and effective management of the treatment process [44,45].

##### Detection of Fungal Infections

PCR also makes a significant contribution to the diagnosis of fungal infections. In particular, the ability of PCR to provide rapid and sensitive results in diagnosing invasive fungal infections allows for timely initiation of antifungal treatments. The detection of clinically important fungi such as *Candida* and *Aspergillus* species can take weeks with traditional methods, whereas PCR enables diagnosis within a few hours. This is especially crucial for immunocompromised patients, as early detection of invasive fungal infections in this group significantly reduces mortality rates [46].

Moreover, one of the broad applications of PCR is the identification of antifungal resistance genes. In cases where pathogens such as *Candida albicans* and *Aspergillus fumigatus* have developed resistance to antifungal treatments, PCR-based tests enable the rapid detection of resistant isolates, guiding the clinical treatment process [47,48].

##### Detection of Parasitic Infections

The effectiveness of PCR in diagnosing parasitic infections is particularly evident in cases where traditional microscopic diagnostic methods are inadequate. Since PCR can directly detect parasite DNA, it is especially effective in identifying asymptomatic or low-intensity infections.

For example, in the diagnosis of malaria caused by *Plasmodium* species, PCR can provide much more sensitive results compared to traditional microscopy methods. The specific detection of malaria species such as *Plasmodium falciparum* is important for clinical management, and PCR can expedite this process [49]. Additionally, the identification of drug-resistant parasites is also possible with PCR [50].

In parasitic diseases such as leishmaniasis, PCR greatly facilitates the diagnostic process. In cases where microscopic diagnosis is challenging, such as with cutaneous and visceral leishmaniasis, PCR can amplify the pathogen’s genomic material to provide a rapid and accurate diagnosis [51,52].

### 2.2. Loop-Mediated Isothermal Amplification (LAMP)

The identification of nucleic acids using amplification strategies has been effectively utilized in diagnostic methods for the accurate detection and identification of causal organisms in the treatment of various infectious diseases. The gold standard for nucleic acid-based diagnostic applications is the PCR, which exponentially and specifically amplifies target DNA sequences [53].

**Figure 2:**
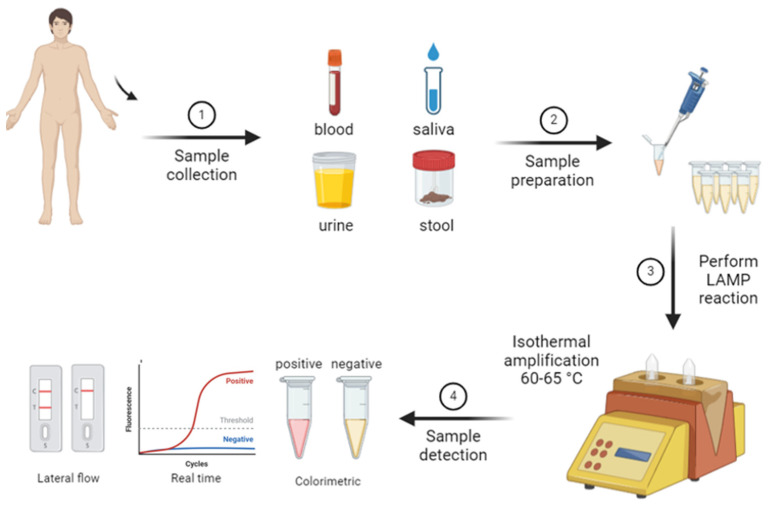
Workflow of loop-mediated isothermal amplification (LAMP) technique.

In recent years, alternative molecular diagnostic methods have been developed in addition to the advantages offered by PCR. These methods overcome some of the limitations of PCR, providing faster, more cost-effective, and portable solutions. One such innovative method is LAMP. LAMP is a rapid and sensitive molecular diagnostic technique that can amplify DNA or RNA at a constant temperature. Unlike PCR, the LAMP method does not require a thermal cycler and is carried out under isothermal conditions, meaning at a single constant temperature. Typically operating between 60 and 65 °C, LAMP delivers quick results and is notable for its ease of use in field settings (Figure 2) [54].

#### 2.2.1. Principles of the LAMP Method

Loop-mediated isothermal amplification (LAMP) is an innovative molecular technique for nucleic acid amplification that operates at a constant temperature, eliminating the need for complex and expensive thermal cyclers [54]. This characteristic offers significant advantages for applications in resource-limited settings and fieldwork. The core principle of LAMP involves the use of a set of four to six primers that bind to six to eight distinct regions on the target DNA sequence, ensuring high specificity [54].

The primer set in LAMP consists of two outer primers (F3 and B3), two inner primers (forward inner primer, FIP, and backward inner primer, BIP), and optional loop primers (loop forward, LF, and loop backward, LB) to accelerate the reaction [54,55]. The inner primers initiate amplification by hybridizing to specific regions on the target DNA, while the outer primers facilitate strand displacement, releasing single-stranded DNA that serves as a template for further amplification. This process results in the formation of stem–loop DNA structures, which are essential for exponential amplification [53].

A key component of the LAMP reaction is the *Bst*. DNA polymerase, derived from *Bacillus stearothermophilus*. This enzyme possesses strong strand-displacement activity and lacks 5′ to 3′ exonuclease activity, preventing the degradation of newly synthesized DNA strands [56]. Its ability to unwind DNA strands without the need for thermal cycling allows the amplification process to occur under isothermal conditions.

The major components required for the LAMP reaction include nucleotides, *Bst*. DNA polymerase, the specific primer set, and a reaction buffer containing magnesium ions [53]. Magnesium ions are crucial for enzyme activity and the stability of the DNA structures formed during amplification.

During the LAMP reaction, the amplification of the target DNA leads to the accumulation of magnesium pyrophosphate, a byproduct that causes turbidity in the reaction mixture. This turbidity can be observed with the naked eye, allowing for simple visual detection of the results [57]. Additionally, LAMP reactions can be monitored using fluorescent dyes [58] or colorimetric indicators [59], enabling real-time observation and easy interpretation of outcomes.

Since its introduction by Notomi et al. in 2000 [54], the LAMP technique has undergone significant advancements, broadening its applications in molecular amplification. Various forms of LAMP assays have been developed to enhance detection capabilities for microorganisms. These include conventional LAMP, reverse-transcription LAMP (RT-LAMP) for amplifying RNA targets, multiplex LAMP for simultaneous detection of multiple targets, and other specialized LAMP formats [60]. Each of these adaptations has contributed to making LAMP a more versatile and powerful tool in the field of molecular diagnostics.

#### 2.2.2. Advantages and Applications of LAMP

Over the past two decades, LAMP has gained enormous popularity as a rapid and cost-effective detection method for numerous infectious diseases. It has demonstrated effectiveness in detecting a broad range of pathogens, from simple bacteria like *Escherichia coli* [61] to emerging viruses such as SARS-CoV-2 [62].

One of the main advantages of LAMP is its ability to deliver rapid results. While PCR typically requires a process lasting 1–2 h, LAMP results can usually be obtained within 30 min to 1 h. This increased speed is due to the isothermal nature of the reaction, which operates at a constant temperature and eliminates the need for cyclic temperature changes required in PCR. This makes LAMP highly suitable for use in field conditions and allows for easy application with portable devices [63].

In terms of cost-effectiveness, LAMP is more advantageous than PCR because it does not require expensive thermal cyclers. The technique can be performed with simple equipment, making it particularly appealing in regions with limited resources [64]. Additionally, the ability to visually detect the byproducts produced during the LAMP reaction eliminates the need for costly detection devices [65]. These characteristics make LAMP an ideal option for low-cost, portable, and rapid diagnostic tests, especially for detecting outbreaks and infections prevalent in tropical regions [66].

LAMP’s utility and efficacy have been well demonstrated in the diagnosis of neglected tropical diseases (NTDs), a group of around 20 diseases caused by bacteria, viruses, fungi, or parasites, mostly prevalent in the poorest regions of the world. Due to poor resources and limited laboratory settings in these areas, LAMP has proven to be a revolutionary technique for therapy monitoring, early disease diagnosis, and contact tracing [67,68].

The method has been effectively applied in diagnosing infections common in tropical regions, such as malaria [69], dengue [70], and Zika virus [71]. In the detection of *Plasmodium* species—the causative agents of malaria—LAMP provides much faster and more reliable results compared to traditional microscopic methods [72]. RNA viruses like dengue and Zika can also be easily detected with LAMP following a reverse transcription step, expanding its utility in virology [70,71].

During the COVID-19 pandemic, LAMP attracted significant attention as an alternative to PCR for detecting the SARS-CoV-2 virus. Its ability to deliver results in a shorter time frame contributed to the widespread adoption of rapid diagnostic tests. LAMP was used to detect the virus in both symptomatic and asymptomatic individuals, and its low cost made it a preferred choice for mass screening efforts. These attributes have established LAMP as an important diagnostic tool for managing pandemics, especially in regions with limited resources [57,64,73].

Although LAMP is a robust and versatile amplification technique, it does have certain limitations. The use of multiple primers increases the risk of non-specific amplification, which can lead to false-positive results [74]. This complexity also makes primer design more challenging for researchers [75]. Moreover, LAMP is less effective for amplifying short gene sequences [76], and achieving multiplexing—simultaneous detection of multiple targets—in a single tube remains a significant hurdle [77].

To address these challenges, significant advancements have been made in LAMP technology. Researchers have focused on optimizing primer design and modifying DNA polymerases to enhance the specificity and efficiency of the reaction. Innovations such as the development of new primer sets and the use of high-fidelity polymerases have improved the accuracy of LAMP assays. Additionally, integrating LAMP with advanced molecular techniques and biosensors has expanded its capabilities. For instance, combining LAMP with lateral flow assays or fluorescent probes allows for more sensitive and specific detection, making it a more versatile and powerful tool for nucleic acid detection [78]. These enhancements have broadened the applicability of LAMP in various fields, from clinical diagnostics to environmental monitoring.

#### 2.2.3. Comparison of LAMP with Other Molecular Methods

Compared to PCR, LAMP has several advantages, including simpler technology and the ability to operate under isothermal conditions, making it suitable for rapid diagnostics without the need for complex laboratory equipment. However, a notable limitation of LAMP is its susceptibility to non-specific amplification, which can reduce its diagnostic accuracy, particularly in complex clinical samples. A systematic review focused on COVID-19 diagnostics found that the pooled sensitivity and specificity of LAMP were 92% and 99%, respectively, while PCR achieved a slightly higher sensitivity of 96% and a specificity of 100% [79]. Moreover, unlike PCR, it does not require complex laboratory equipment, making it easier to implement in the field. The low cost and speed of LAMP make it particularly valuable in resource-limited regions and during outbreaks requiring urgent diagnosis [57,78].

However, LAMP does have some limitations. The primer design for LAMP is quite complex, and the selection of appropriate primers plays a critical role in the success of the method. Additionally, while LAMP is typically optimized for the detection of a single pathogen, PCR has the capability to detect multiple targets simultaneously. Therefore, LAMP is generally used for the detection of single-pathogen infections, and in clinical samples containing multiple pathogens, PCR may be more advantageous than LAMP [78,80].

### 2.3. Nucleic Acid Sequence-Based Amplification (NASBA)

Nucleic acid sequence-based amplification (NASBA) was developed by Compton in the early 1990s [81]. Unlike PCR, NASBA operates under isothermal conditions, amplifying nucleic acids without the need for temperature cycling. NASBA involves the conversion of RNA into complementary DNA (cDNA) by reverse transcriptase, followed by RNA synthesis from the cDNA template via RNA polymerase. The newly synthesized RNA then serves as a template for further amplification in subsequent NASBA cycles [82].

The NASBA reaction is conducted at approximately 41 °C and can achieve up to a 10^9^-fold amplification within 90 min without requiring a thermocycler. This amplification process relies on three enzymes—avian myeloblastosis virus reverse transcriptase, T7 RNA polymerase, and RNase H—and two primers, P1 and P2. Primer P1 contains a binding site for T7 RNA polymerase, facilitating RNA synthesis from the RNA-DNA hybrid with the assistance of reverse transcriptase. Once the RNA is removed from the hybrid, RNase H degrades it, allowing the remaining cDNA to bind with P2, which assists in synthesizing complementary strands. The presence of reverse transcriptase makes NASBA highly effective for RNA amplification [80,83].

RNA detection with NASBA can be performed using various methods, such as the dideoxy method (involving reverse transcriptase and a labeled oligonucleotide primer), enzyme-linked gel assay (ELGA), electrochemiluminescent detection, molecular beacon detection, southern hybridization, and fluorescent correlation spectroscopy [80,81].

Several commercial NASBA kits are available, produced by companies such as Qiagen, Inc. (Valencia, CA, USA), Life Sciences, Inc. (St. Petersburg, FL, USA), KIT Biomedical Research (Meibergdreef, Amsterdam, The Netherlands), Gen-Probe, Inc. (San Diego, CA, USA), and bioMérieux, Inc. (Durham, NC, USA) [84].

### 2.4. Sequencing Technologies

**Figure 3:**
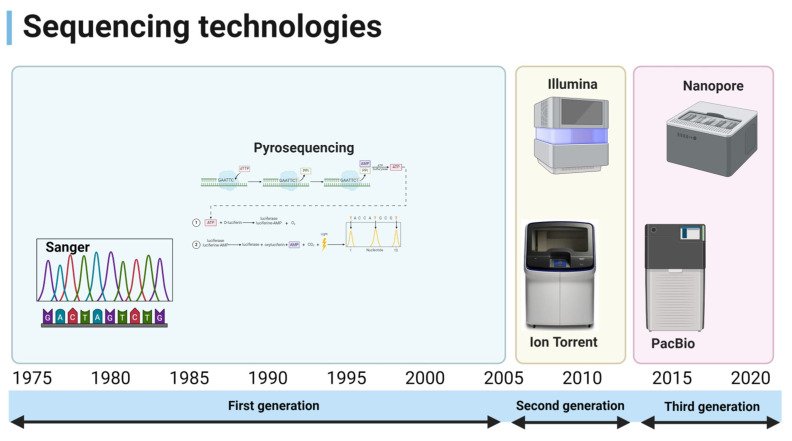
Generations of sequencing technologies.

The advancement of sequencing technologies has significantly transformed the field of genomics, introducing various generations of methods with distinct capabilities and applications. These technologies are broadly categorized into first-generation, second-generation (next-generation sequencing), and third-generation sequencing techniques. While Sanger sequencing represents the first generation with its high accuracy for small DNA fragments, the second and third generations have brought substantial improvements in speed, throughput, and cost-effectiveness (Figure 3).

#### 2.4.1. Sanger Sequencing Technology

Sanger sequencing uses certain nucleotides with end chains (dideoxy nucleotides) which do not have a 3′-OH group. As a result, DNA polymerase is unable to form a phosphodiester bond, causing the growing DNA chain to terminate at that point. The ddNTPs are fluorescently or radioactively labeled for detection in automated sequencing devices [85]. Sanger sequencing remains a common approach for sequencing small DNA sequences since it yields high-quality DNA sequences up to 1000 bases.

The primary application of Sanger sequencing technology encompasses single-reaction sequencing operations that utilize a specified DNA primer paired with a given template, commonly employed for validating plasmid constructs and polymerase chain reaction products. This technique relies on low-cost molecular biological products, such as DNA purification reagents and kits, as well as manufactured primers of high quality and commercial accessibility [86]. Additionally, Sanger sequencing can be utilized to assess the functional properties of specific enzymes on fluorescently labeled DNA substrates by examining DNA fragment size. Various fluorescent labels, substrates, products, and reaction intermediates can be evaluated through capillary electrophoresis in a single experimental framework. This approach enables the detection of enzymatic processes including DNA polymerase and DNA ligase kinetics, as well as coupled enzyme processes such as the processing of Okazaki fragments and ribonucleotide excision repair. High-throughput experiments have also been conducted utilizing capillary electrophoresis [87].

#### 2.4.2. 16S rRNA Gene Sequencing

Ribosomal RNA (rRNA) is essential for the proper assembly of the ribosome, the enzyme that synthesizes cellular proteins. The small ribosomal subunit’s 16S rRNA could be used to distinguish various taxonomic groups. Hence, this became the primary strategy for classifying bacteria into phylogenetic groups and has been used for bacterial taxonomy research for over 40 years. A highly effective method for accurate pathogen identification is the application of 16S rRNA gene sequencing which, being present in all bacteria, is considered the “gold standard” in the field of bacterial identification [88]. Currently, many bacteria are classified with 16S rRNA using specific primers. The key principles of this method include primer of the gene selection, PCR amplification of the selected gene, sequencing of amplified DNA, data analysis using bioinformatic tools, and comparative analysis [89].

The application of 16S rRNA sequencing for pathogen identification serves several advantages over traditional methods. The utilization of 16S rRNA sequencing for the identification of pathogens presents numerous benefits compared to conventional techniques. Sequence analysis of the 16S rRNA gene allows for the differentiation of organisms at the genus level across all major phyla of bacteria, as well as the classification of strains at multiple taxonomic levels, including species and subspecies associated with health and disease states [90]. Furthermore, 16S rRNA sequencing can be specifically useful in identifying unknown isolates or those with ambiguous biochemical profiles [91]. However, in the application of 16S rRNA sequencing, there are some limitations such as limited resolution for closely related species, potential PCR biases, variability in 16S rRNA copy numbers among different bacterial species, and the fact that it is time-consuming and costly [91,92].

#### 2.4.3. Next-Generation Sequencing (NGS)

Next-generation sequencing (NGS) technologies have changed the face of genomics by providing high throughput, rapid, and cost-effective DNA sequencing compared to traditional Sanger sequencing methods. This technology allows for the simultaneous sequencing of numerous DNA and RNA molecules, enabling the high-capacity, sensitive, and rapid analysis of genetic material, breaking new ground in the molecular diagnosis of infectious diseases [93]. In addition to providing comprehensive genome sequencing, NGS has also facilitated transcriptomic, epigenomic, metagenomic, and other omics studies [94].

The fundamental principle of NGS is its ability to read billions of base sequences in parallel. This technology allows for the analysis of the genetic material of specific pathogens as well as the detection of various infectious agents such as viruses, bacteria, and parasites. It also offers high efficiency in identifying genetic variations and mutations, and even discovering new pathogen species [93]. One of the key features that sets NGS apart from other molecular methods is its ability to analyze the entire microbiome with a single test. However, the implementation of NGS is a costly process that requires technical infrastructure, which limits its widespread use in clinical laboratories. Nonetheless, the development of portable NGS devices and the reduction in costs are expected to make this technology more widely accessible in the near future [95].

##### NGS Platforms

Next-generation sequencing technologies have transformed genomics by offering rapid, cost-effective, and high-throughput DNA sequencing compared to traditional methods like Sanger sequencing [96]. This diversity of platforms has facilitated a wide range of applications, including genomic, transcriptomic, and proteomic research as well as metagenomic and microbial diversity studies [97]. Oxford Nanopore, Ion Torrent, Pacific Biosciences, and Illumina are some of the leading next-generation sequencing platforms. The Illumina and Ion Torrent platforms are based on the principle of sequencing by synthesis, while Pacific Biosciences and Oxford Nanopore use single-molecule real-time sequencing and nanopore sequencing, respectively [96]. Because each platform has unique read length, accuracy, throughput, and cost advantages and disadvantages, they can be used for a variety of applications and research objectives [98].

Ion Torrent amplifies adaptor-ligated DNA fragments on the surface of beads using emulsion-PCR. After that, the beads are put in microwells to undergo the sequencing-by-synthesis process. It uses luciferase-based light production to identify nucleotides. The pH variations brought on by the release of hydrogen ions during DNA extension are measured by Ion Torrent’s semiconductor sequencing. An ion sensor in the microwells detects these pH variations and converts them into a voltage signal that is proportionate to the number of bases present [99]. Ion Torrent can provide a high throughput of nearly 10 gigabases in just 2 to 6 h and enables a larger number of samples to be run in parallel. The ability to identify the pathogens is one of this sequencer’s key benefits [100,101].

The widespread adoption of Illumina’s NGS technologies stems from several key factors, including their notable innovations and the availability of a diverse array of equipment options. Firstly, these systems provide high precision in sequencing, ensuing credible outcomes. Secondly, the cost-effectiveness of these methods, measured in terms of gigabytes of data generated, facilitates researchers in selecting the most appropriate tools tailored to the pecuniary requirements and scope of their specific projects. The range of sequencing machinery encompasses compact bench-top devices, such as the MiniSeq, featuring moderate performance, and extensive, large-scale instruments, such as the HiSeqX, which enables the sequencing of entire genomes within population-based initiatives [102]. Clonal amplification of DNA fragments ligated to adaptors on a glass slide, referred to as bridge amplification, is the first step in the sequencing process. Fluorescent nucleotides that have been incorporated are read using a cyclic reversible termination technique. Fluorescently labeled nucleotides are incorporated, washed, imaged, and cleaved to sequence the template strand nucleotide by nucleotide. Fluorescently labeled 3′-O-azidomethyl-dNTPs are used to break up the nucleotide incorporation, which enables the removal of unincorporated bases and the identification of the added nucleotide through fluorescence imaging [103].

The ability to sequence incredibly long DNA and RNA molecules makes third and fourth generation sequencing techniques—also referred to as long-read technologies—a drastic divergence from previous sequencing techniques. The third generation does not require library preparation of the DNA template for sequencing and can produce read lengths greater than 10 kb. Two leaders in this area are Oxford Nanopore Technology (ONT) and Pacific Biosciences (PacBio) [104]. PacBio Sequencing allows for the long-read sequencing of DNA fragments up to tens of kilobases in length by using a single-molecule real-time (SMRT) technique with fluorescently labeled nucleotides. A high-throughput platform, the PacBio sequencer stands out from other sequencing platforms due to its capacity to produce full-length gene sequences [105]. Being able to completely identify and structurally analyze complex microbial communities makes PacBio a useful tool. The ability to detect DNA alterations in real time during SMRT without the need for previous amplification makes it a valuable tool in epigenetic research as well [106].

The Nanopore MinION^TM^ sequencer, the most recent NGS technology, was created by Oxford Nanopore Technologies in 2014. Oxford Nanopore Technology utilizes the process of single-molecule sequencing. The technology of nanopore sequencing relies on the observation that single-stranded DNA (ssDNA) experiences a shift in electric charge as it passes through a protein channel, known as a nanopore. The enzyme DNA polymerase enables the passage of ssDNA through a nanopore, and this technology detects ssDNA molecules according to the characteristic electrical current changes in bases [107]. More than 50 kbp of DNA can be sequenced using Oxford Nanopore Technology. Actually, Nanopore’s ability to quickly prepare libraries and acquire genomic data in real time—two important features that distinguish it from NGS platforms—is likely the greatest advancement in DNA sequencing that has been made [108]. There are now countless applications for this technology in every environmental condition. This is due to the fact that it is portable, does not require any complex equipment for library preparation, and enables on-site analysis using just a computer and no bulky laboratory equipment [109].

##### The Use of NGS in Diagnosis and Treatment

NGS technology plays a significant role not only in detecting pathogens but also in identifying antibiotic resistance and virulence factor genes [110]. For example, the detection and monitoring of resistance genes in pathogens such as carbapenem-resistant *Klebsiella pneumoniae*, a bacterium carrying antibiotic resistance, is possible with NGS technology [111]. This is crucial for controlling hospital-acquired infections and tracking the spread of antibiotic resistance. Additionally, NGS enables the epidemiological monitoring of pathogens and understanding their spread dynamics, which plays a critical role in planning public health measures [112].

Especially during outbreaks, the genetic information provided by NGS offers rapid and comprehensive analysis, contributing to the shaping of public health policies. The interest in using this technology during the COVID-19 pandemic was particularly notable for tracking genetic mutations and different variants of the virus. NGS became an essential tool for understanding the evolution of SARS-CoV-2 and the spread of variants in different regions [113]. Additionally, this information played a critical role in vaccine development efforts and public health measures [114]. The emergence of variants such as Delta, Beta, and Omicron highlighted the vital importance of NGS in monitoring mutations [115].

### 2.5. Metagenomic Approaches and Clinical Applications

One of the innovations brought by NGS technologies is the use of metagenomic approaches in clinical applications. Metagenomics allows for the sequencing of all microbial genomes present in environmental samples or disease tissues, enabling the analysis of complex microbial communities without the need to detect individual pathogens. This technique is particularly useful for identifying pathogens that cannot be cultured by traditional methods or are previously unknown. Clinical metagenomics serves as an important diagnostic tool for rare pathogens or newly emerging disease agents [116].

Metagenomic approaches offer a non-hypothesis-driven screening method. This allows for the sequencing and analysis of all microbial genomes present in a sample without prior knowledge of the infection’s cause. Such an approach provides a significant advantage in resolving complex or unknown infections. For example, metagenomic analysis is one of the most effective methods for diagnosing conditions like meningitis or sepsis when the cause cannot be determined. Additionally, it plays a crucial role in outbreak management and individual patient care by providing information on the origins and transmission pathways of diseases [116,117,118].

However, it is important to select the right platform and carefully consider factors such as sample input amount, read length, cost, and coverage [119]. One of the problems with these platforms is bioinformatic analysis because it is hard to distinguish which sequences correspond to a certain microorganism in the data set. The reason for this challenge is that most of the sequences belong to the host. The bioinformatic process comprises de novo assembly, binning, functional and taxonomic analysis, and fragment recruitment to reference genomes [120]. Several databases provide information during the identification of viruses or other microorganisms. The most used one for metagenomics analysis is The National Center for Biotechnology Information (NCBI) [121]. Besides NCBI, several databases such as the Reference Viral Database (RVDB) (https://rvdb.dbi.udel.edu/, accessed on 10 November 2024) and MEGARes (https://www.meglab.org/, accessed on 10 November 2024) are available for identifying organisms or antimicrobial resistance genes.

Metagenomic applications provide important insights not only into infectious diseases but also into immune responses and pathogen–host interactions by studying the human microbiome. For example, metagenomic analyses in infectious diseases can reveal how pathogens and host defense mechanisms interact, thereby aiding in the development of more personalized treatment approaches [122].

A substantial body of research has investigated the potential utilization of multiplex next-generation sequencing (mNGS) across diverse clinical contexts. A study conducted to detect the pathogens in pediatric oncology patients with suspected bloodstream infections (BSI) revealed that in comparison to established reference tests, mNGS exhibits superior sensitivity and clinical concordance in identifying putative pathogens and distinguishing between BSI and non-BSI, with notable efficacy in the clinical detection of viruses [123]. mNGS has recently been utilized to identify microorganisms from central nervous system infections [124,125], pulmonary infection [126], meningitis and encephalitis [127], and febrile patients with suspected infection [128].

On the other hand, some researchers argue that targeted next-generation sequencing (tNGS) is more effective than mNGS. This limitation arises from the high cost of mNGS for integrated DNA and RNA analysis and its relatively constrained capacity to extract pathogen genome information. Therefore, mNGS is difficult to apply in repetitive and sustained disease surveillance [129]. However, tNGS requires the development of custom primers for prespecified pathogens, facilitating the construction of tailored diagnostic panels. This technique has been shown to be cost-effective, with excellent specificity, minimal sample requirements, and the ability to avoid human DNA contamination [130]. tNGS has shown significant potential not only in diagnosing tuberculosis [131,132] and respiratory pathogens [133] but also in identifying a wide range of other infectious agents in clinical settings.

Although there are various sequencing-based diagnostic techniques that test for many microorganisms simultaneously, these tests require samples from infected tissue, hence limiting the types of pathogens to those from the tissue [134]. As metagenomic plasma microbial cell-free DNA (mcfDNA) sequencing is a wide, unbiased, noninvasive diagnostic tool that has the potential to improve infection diagnosis over current testing procedures that rely on a battery of microbiologic and molecular tests [135]. This sequencing technique has emerged as an effective method for detecting and quantifying microbial populations in a variety of biological materials, including plasma [136]. This methodology offers several strengths, including its ability to provide a comprehensive and unbiased assessment of the microbial landscape without requiring prior knowledge of specific microbial markers.

One of the primary advantages of plasma mcfDNA sequencing is the ability to diagnose and monitor diseases without intrusive procedures. Studies have demonstrated that the presence and abundance of certain microbial species in the blood can be associated with various disease states, ranging from infectious diseases to cancer and autoimmune disorders [137,138].

### 2.6. The Future of NGS and Metagenomics

The widespread use of NGS in clinical applications is currently limited due to high costs and technical infrastructure requirements. However, this situation is beginning to change with the rapid development of portable NGS devices. The advancement of portable NGS technology and the reduction in sequencing costs are significant developments that will enable more widespread use of this technology in clinical laboratories [95].

In the future, as NGS and metagenomic approaches become more cost-effective and faster, they are expected to become standard methods for diagnosing infectious diseases. The role of these technologies will especially grow in the fields of personalized medicine and pathogen monitoring, providing effective results even in more complex clinical samples.

In addition to its advantages in diagnosing infectious diseases, NGS has important applications in areas such as monitoring viral genomic variations, expediting vaccine development, and tracking gene transfer between pathogens [139]. This plays a crucial role in controlling outbreaks and determining treatment strategies [140].

### 2.7. CRISPR-Based Diagnostic Methods

Another innovative approach is diagnostic methods based on clustered regularly interspaced short palindromic repeats (CRISPR) and CRISPR-associated protein (Cas) technology. Although CRISPR-Cas technology is primarily known as a gene-editing tool, it has also been successfully used for the specific detection of genetic material. This method stands out for its ability to deliver rapid, sensitive, and specific results [141].

Bacteria and archaea use CRISPR systems as adaptive immune systems to defend against plasmids and viruses that invade their environments. Fundamentally, CRISPR systems are a vital part of a microbial immune system that uses endonuclease activity connected to the Cas enzyme to eliminate the invasive pathogen after identifying foreign nucleic acids based on their sequence [142]. The system works through a two-step process: acquisition and interference. During acquisition, fragments of viral DNA are integrated into the host genome as CRISPR sequences. Upon subsequent infections, the CRISPR sequences are transcribed and processed into CRISPR RNAs (crRNAs) that guide the Cas proteins to complementary viral sequences, leading to cleavage and inactivation of the invader [143,144].

CRISPR-Cas systems are classified in two groups based on Cas protein. Class 1 consists of type I (cas3), type III (cas10), and type IV (unknown function). Class 2 CRISPR-Cas systems comprise type II (Cas9), type V (Cas12), and type VI (Cas13). While class 1 uses multi-Cas protein complexes for function, class 2 has a single effector protein [145]. Endonuclease enzymes Cas9 and Cas12 can cut double-stranded DNA (dsDNA), while Cas13 targets RNA, unlike Cas9, which becomes inactive after target cleavage. Cas12 and Cas13’s nuclease enzymes can cleave both target and non-target sequences after they have targeted desired sequences. These enzymes are therefore activated and will begin cutting continuously if the target sequence is present in a sample [146]. Cas9, Cas12, Cas13, and Cas14 are the most commonly used enzymes in CRISPR-based detection systems, all of which belong to the class 2 category [147]. The effectiveness of CRISPR-based therapies is fundamentally dependent on the safety and performance of Cas proteins [148].

In 2016, Pardee et al. developed CRISPR-Cas systems to identify nucleic acids for molecular diagnostics for the first time [149]. DNA Endonuclease-Targeted CRISPR Trans Reporter (DETECTR) diagnostic tool was presented in 2017 by Jennifer Doudna’s team to detect Human Papillomavirus (HPV). This approach is based on the Cas12a activity [150]. In 2018, Zhang group introduced Specific High Sensitivity Enzymatic Reporter UnLOCKing (SHERLOCK) to detect viruses such as Zika and Dengue. SHERLOCK is a diagnostic tool that utilizes CRISPR/Cas13 to detect RNA viruses, offering a rapid and highly sensitive method for identifying pathogens in clinical samples [151]. This technique targets RNA rather than DNA using the Cas13 enzyme, which functions as a ribonuclease (RNase). When the Cas13 enzyme attaches to RNA targets through gRNAs, it becomes active. Quantifiable signals are produced when activated Cas13 cleaves RNA target sequences and non-target reporters surrounding ssRNA [152]. COVID-19 infection has been reported to be frequently asymptomatic. Therefore, rapid screening of infected individuals was necessary during the pandemic. In this process, the DETECTR system focused on detecting the presence of SARS-CoV-2-specific mutations in the N and E genes. When both genes are present, a positive result is produced, and the technique has been refined to eliminate false positives from other coronaviruses [153]. In contrast, the SHERLOCK method gives a positive result if the S and Orflab gene sequences are present [152].

FNCAS9 editor limited uniform detection assay (FELUDA) is another platform used for CRISPR based diagnosis. A Cas9 enzyme derived from *Francisella novicida* (FnCas9) exhibits remarkable precision, demonstrating minimal interaction with mismatched substrates; however, its limited efficiency in cellular targeting poses a significant constraint on its therapeutic application [148,154].

The use of CRISPR-based biosensors is a method for identifying and quantifying targeted molecules in complex samples. A recognition component that interacts with the target molecule and a transducer that transforms the binding event into a measurable signal are the usual components of these biosensors. CRISPR biosensors use the Cas protein and crRNA to selectively identify and bind to the targeted molecule. After the binding process, the Cas protein may change in ways that produce a signal that can be detected, like fluorescence or enzymatic activity [155]. This signal is quite sensitive because it can be measured and quantified. Hence, the emergence of CRISPR biosensors has opened up significant opportunities in areas such as disease diagnosis.

The application of CRISPR/Cas technology to pathogen identification marks a substantial improvement in diagnostics. Rapid and accurate identification of infectious diseases allows for timely interventions, lowering transmission rates and increasing patient outcomes. Furthermore, the ability to quickly identify new infections can improve surveillance and response techniques during outbreaks, eventually contributing to global public health initiatives. In the future, CRISPR-based diagnostic systems are expected to develop further and become more widespread. Additionally, the impact of CRISPR technology is increasing in personalized medicine and the diagnosis of genetic diseases. The flexibility offered by CRISPR-Cas systems will allow this technology to be integrated into new diagnostic platforms and find a broader range of applications.

## 3. Future Perspectives and Challenges

### 3.1. The Future Role of Molecular Diagnostic Methods

In the future, the integration of artificial intelligence (AI) and machine learning (ML) technologies into the analysis of molecular diagnostic data will make diagnostic processes faster and more accurate. Especially in the analysis of NGS and metagenomic data, where large amounts of data are generated, AI and ML algorithms can contribute in the following ways:

- Automation of Data Analysis: by using artificial intelligence in the analysis of large-scale genetic data, it will be possible to rapidly detect and classify pathogens. This significantly reduces diagnostic time and minimizes human error.
- Detection of Resistance Genes and Mutations: AI-assisted analyses increase the accuracy in identifying antibiotic resistance and genetic variations in pathogens, enabling more precise results. This allows for the rapid determination of appropriate treatment options.

### 3.2. The Role of Artificial Intelligence and Machine Learning

The use of molecular diagnostic methods in clinical laboratories enables diagnostic processes to be carried out more rapidly and accurately. In the future, advanced technologies such as PCR, NGS, and CRISPR are expected to become more widespread and accessible. As these technologies become standard practices in healthcare, significant advancements are anticipated in the following areas:

- Widespread Use of Rapid Diagnostic Tests: the development of portable and low-cost molecular diagnostic devices will enable the expansion of rapid diagnostic tests in the field and rural areas. This provides a critical advantage, particularly for the early detection of outbreaks and controlling the spread of infectious diseases.
- Personalized Medicine Applications: molecular diagnostic technologies will support personalized medicine approaches, such as genetic profiling and the identification of individual disease risks, allowing for more effective treatment planning.

### 3.3. Clinical and Ethical Challenges

The widespread use of molecular diagnostic methods also brings certain clinical and ethical challenges.

- Cost and Infrastructure Requirements: the need for high-cost equipment and technical infrastructure limits the use of these technologies in low- and middle-income countries. In developing countries, innovative solutions should be developed to reduce costs and promote the widespread use of portable devices.
- Detection of Resistance Genes and Mutations: comprehensive genetic analyses such as NGS can result in the acquisition of sensitive information about individuals’ genetic data. This raises ethical concerns and issues related to data security. Therefore, legal regulations should be developed to ensure the protection and ethical use of genetic data.

### 3.4. Development of Portable and Low-Cost Diagnostic Devices

In recent years, the development of low-cost molecular diagnostic systems, such as portable PCR and NGS devices, has made it possible to conduct diagnostics in field conditions. These innovations will play a significant role, especially in monitoring and controlling infectious disease outbreaks. In the future, the widespread adoption of such devices and further reductions in costs are expected.

### 3.5. The Future of CRISPR-Based Diagnostic Systems

The use of CRISPR technology for diagnostic purposes holds great potential, particularly for the rapid and sensitive detection of DNA and RNA targets. In the future, CRISPR-based diagnostic systems are expected to further develop and become more widespread. These innovative diagnostic methods could have a significant impact in the following areas:

- Personalized Medicine: diagnostic and treatment approaches tailored to patients’ genetic profiles could be offered;
- New Diagnostic Platforms: CRISPR-based systems can enable the development of portable and low-cost diagnostic devices, providing ideal solutions for rapid diagnostics in field conditions.

### 3.6. Perspective on the Integration of Molecular Diagnostic Techniques in Low- and Middle-Income Countries

As authors from Turkey, Nigeria, and Libya, we are aware of the distinct challenges and opportunities involved in implementing molecular diagnostic technologies, such as PCR, NGS, and LAMP, in low- and middle-income countries. While the initial costs and infrastructure requirements of these advanced techniques may be significant, their potential to improve infectious disease management and public health outcomes makes them essential for sustainable healthcare development.

For instance, during the COVID-19 pandemic, many low- and middle-income countries successfully expanded molecular diagnostic capacities through strategic investments and partnerships. In Nigeria, the number of public health laboratories capable of molecular testing was increased from four to seventy-two within a year, demonstrating the scalability of these technologies even in resource-limited settings [156]. In Turkey, where laboratory infrastructure is relatively advanced, integrating molecular diagnostics might focus on establishing regional testing centers and expanding healthcare personnel training to enhance diagnostic capabilities. Meanwhile, in countries like Libya and Nigeria, where resource limitations are more pronounced, implementing portable and cost-effective diagnostics such as LAMP could offer practical solutions to improve access. Collaborations with global health organizations and support from local governments can play a crucial role in this process.

Furthermore, sustainable integration can be supported by using locally sourced materials where feasible, simplifying diagnostic workflows, and creating targeted training programs for healthcare professionals. These strategies would not only enhance access to essential diagnostic tools but also strengthen local public health systems, enabling timely responses to outbreaks and improving disease surveillance. Through these efforts, molecular diagnostics can play a transformative role in advancing healthcare outcomes in low- and middle-income countries, even in the face of economic constraints.

## 4. Conclusions

Molecular diagnostic methods have significantly advanced the diagnosis and management of infectious diseases, offering rapid, sensitive, and specific detection capabilities that surpass traditional techniques. The emergence of technologies such as PCR, NGS, and CRISPR has transformed clinical microbiology by enabling the early identification of pathogens, detection of antimicrobial resistance, and monitoring of genetic variations. As these methods continue to evolve, they are becoming more accessible and practical, with the development of portable, low-cost diagnostic devices expanding their use in resource-limited settings. However, the high costs and infrastructure requirements associated with these technologies, along with the need for skilled personnel, still pose challenges, particularly in low- and middle-income countries. Innovative solutions to reduce costs and simplify the technology are essential to overcoming these barriers and promoting global health equity.

Future directions in molecular diagnostics will likely involve the integration of artificial intelligence and machine learning for the automated analysis of complex datasets generated by technologies such as NGS and metagenomics. This approach could significantly enhance the accuracy and speed of diagnostic processes, while also aiding in the identification of resistance genes and novel pathogens. Moreover, ethical considerations related to genetic data privacy and the use of sensitive information must be addressed through the establishment of appropriate regulatory frameworks. As CRISPR-based diagnostics mature, they could further revolutionize the field, providing personalized medicine solutions and rapid, field-deployable testing for emerging infectious diseases. Together, these advancements hold the promise of more effective disease control, improved patient outcomes, and the potential to transform global health practices.

## Figures and Tables

**Table 1:** Advantages, disadvantages, and clinical applications of molecular diagnostic methods.

Technology	Advantages	Disadvantages	Applications
PCR	High sensitivity, early diagnosis, broad clinical use	Requires thermal cycler, may produce false positives/negatives in some cases	Diagnosis of viral, bacterial, fungal, and parasitic infections
qPCR (Real-Time PCR)	Allows quantitative analysis, simultaneous detection of multiple pathogens	Expensive equipment, requires thermal cycling	Viral load monitoring, tracking disease progression
Multiplex PCR	Detection of multiple pathogens at once	Complex primer design, requires optimization	Respiratory infections, co-infections
LAMP	Isothermal operation, rapid results, portable, and low cost	Difficult primer design, usually targets a single pathogen	Tropical infections, rapid field diagnosis
NASBA	Isothermal amplification, high sensitivity for RNA detection	Requires RNA isolation, limited to specific pathogens	Detection of RNA viruses, tuberculosis, rapid field diagnostics
NGS	Genome scanning, detection of new variants and resistance genes	High cost, requires technical infrastructure and expertise	Antibiotic resistance, outbreak monitoring, discovery of new pathogens
CRISPR	Precise detection of specific DNA/RNA sequences, rapid results	Limited clinical applications so far, technically complex	Detection of viral infections, genetic variants

## Data Availability

No new data were created or analyzed in this study.
